# Interfacing Langmuir–Blodgett and Pickering Emulsions for the Synthesis of 2D Nanostructured Films: Applications in Copper Ion Adsorption

**DOI:** 10.3390/nano14090809

**Published:** 2024-05-06

**Authors:** Andrei Honciuc, Oana-Iuliana Negru, Mirela Honciuc

**Affiliations:** “Petru Poni” Institute of Macromolecular Chemistry, 41A Gr. Ghica Voda Alley, 700487 Iasi, Romania; negru.oana@icmpp.ro (O.-I.N.); teodorescu.mirela@icmpp.ro (M.H.)

**Keywords:** Pickering emulsions, self-assembly of nanoparticles, Langmuir–Blodgett nanoparticle monolayers, photonic monolayers, metal-ion adsorbents, heavy metal ions

## Abstract

This research focuses on developing a 2D thin film comprising a monolayer of silica nanoparticles functionalized with polyethyleneimine (PEI), achieved through a novel integration of Langmuir–Blodgett (L-B) and Pickering emulsion techniques. The primary aim was to create a nanostructured film that exhibits dual functionality: iridescence and efficient metal ion adsorption, specifically Cu(II) ions. The methodology combined L-B and Pickering emulsion polymerization to assemble and stabilize a nanoparticle monolayer at an oil/water interface, which was then polymerized under UV radiation to form an asymmetrically structured film. The results demonstrate that the film possesses a high adsorption efficiency for Cu(II) ions, with the enhanced mechanical durability provided by a reinforcing layer of polyvinyl alcohol/glycerol. The advantage of combining L-B and Pickering emulsion technology is the ability to generate 2D films from functional nanoparticle monolayers that are sufficiently sturdy to be deployed in applications. The 2D film’s practical applications in environmental remediation were confirmed through its ability to adsorb and recover Cu(II) ions from aqueous solutions effectively. We thus demonstrate the film’s potential as a versatile tool in water treatment applications owing to its combined photonic and adsorptive properties. This work paves the way for future research on the use of nanoengineered films in environmental and possibly photonic applications focusing on enhancing the film’s structural robustness and exploring its broader applicability to other pollutants and metal ions.

## 1. Introduction

The fabrication and application of highly organized nanoparticle monolayers and 2D thin films are rapidly expanding fields, with applications ranging from microelectronics to photonic crystals and environmental remediation [1,2,3]. The Langmuir–Blodgett (L-B) techniques are widely known for their use in the preparation of molecular monolayers [4] or nanoparticle monolayers, which are thin films at the air–water interface that can be transferred onto solid substrates. It is known that through the L-B method, the molecules or nanoparticles can be “driven into” an ordered monolayer at the air–water interface by compressing the 2D surface with the help of two surface sweeping barriers. In this way, a plethora of monolayers have been prepared in fundamental studies on organic/unimolecular electronics [5], photonic crystals [6,7], etc. One of the great disadvantages of the L-B methods in the preparation of monolayers based on nanomaterials and nanoparticles is that even though highly ordered monolayers can be prepared at the surface of water [8], this ordering can be severely disrupted upon their transfer onto solid substrates, especially when there is little cohesion in the 2D plane between nanoparticles; thus, some adaptations to the L-B technique [9] are needed to overcome these limitations. 

Recent advances in nanoparticle self-assembly offer promising techniques to overcome these limitations and offer inspiring examples. For example, lately, a variety of techniques have been developed to preserve the order of the self-assembled nanoparticle monolayer at the air–water or liquid–liquid interface, conferring this way more cohesion and rigidity to the monolayer. For example, for constructing highly ordered 2D nanoparticle thin films, strategies such as interfacial assembly and kinetic control techniques [10], the application of an electric field [11], the use of surfactant linkers to promote assembly [12], adjustments to surface charge density [13,14], spreading nanoparticles on the L-B trough from mixtures of high- and low-boiling point solvents [15], etc., have been implemented. Considerable efforts have also been directed towards refining this interfacial assembly process by optimizing the contact angles of nanoparticles to enhance assembly [16], enabling monolayer transfer onto solid surfaces, with the potential for development of new skin-like wearables [17].

In this work, we explore a new approach to the preparation of a highly ordered 2D monolayer of nanoparticles by self-assembly of a monolayer at the liquid–liquid interface on a Langmuir–Blodgett trough utilizing Pickering emulsion polymerization technology. Pickering emulsions are emulsions stabilized by nanoparticles. The stabilization process takes place via irreversible adsorption of a monolayer of nanoparticles at the liquid–liquid interface of an emulsion droplet. Sometimes, these nanoparticles are self-assembled into a highly organized monolayer. 

In this context, we note that Pickering emulsion polymerization technology (PEmPTech) was utilized to “lock in” or “trap” highly organized monolayers through in situ polymerization of dispersed oil emulsion droplets, whereas the oil is a vinyl-bearing monomer immiscible with water, generating surface nanostructure polymer microspheres and thereby preserving the monolayer’s structure [18]. However, there is great unexplored potential in the preparation of highly ordered self-assembled monolayers of nanoparticles through integration of these PEmPTech techniques with L-B methods to enhance film functionality and structural integrity.

Significant research has been conducted on the self-assembly of nanoparticles into monolayers and thin films. Studies have demonstrated that various materials, including silica, gold, and polymeric nanoparticles, can be arranged into structured films via L-B methods with applications in sensors, optical devices, and catalysis [19,20,21]. Yet, the integration of such films into practical applications often requires additional structural enhancement and functionalization, which remains a less explored area. This work aims to bridge this gap by combining the L-B method PEmPTech to create nanostructured 2D polymer thin films with a dual functionality: photonic properties due to the ordered nanoparticle assembly and a metal ion adsorption capacity. We propose that the innovative application of these combined techniques not only preserves the structural integrity of the nanoparticle monolayers but also significantly expands their functional capabilities, particularly in environmental applications. In addition, due to structural ordering, the obtained 2D films exhibit iridescence and a hint of structural colors, which are most commonly found in nature and biomimetic materials [22,23].

## 2. Materials and Methods

### 2.1. Materials

Polyvinyl alcohol (PVA), pellets, (average molecular weight M_w_ = 12.4 × 10^4^ g/mol, degree of hydrolysis DH = 99 ÷ 100%), and glycerin (99.6%) were purchased from Acros Organics (Geel, Belgium). Tert-butyl acrylate (t-BA), 98%, containing 10–20 ppm monomethyl ether hydroquinone as an inhibitor; divinylbenzene (DVB), technical grade 80%, stabilized with the inhibitor monomethyl ether hydroquinone; 3-(triethoxysilyl) propionitrile, 97% (TESPN); aluminum oxide (Al_2_O_3_); tetraethylorthosilicate, 99% (TEOS); N,N′-dicyclohexylcarbodiimide, 99% (DCC); branched polyethyleneimine (average M_w_~25,000 by LS, average M_n_~10,000 by GPC); and anhydrous N,N-Dimethylformamide, 99.8% (DMF), were purchased from Sigma-Aldrich (Merck, KGaA, Darmstadt, Germany). All products containing inhibitors were first purified through an Al_2_O_3_ column prior to their usage.

Pure copper chloride (II) dihydrate p.a. (99.0% CuCl_2_∙2H_2_O) was bought from ChemPUR Feinchemikalien und Forschung GmbH (Karlsruhe, Germany); hydrochloric acid (HCl), ≥37%, was purchased from Fluka (Honeywell Specialty Chemicals, Seelze, Germany); and ethanol absolute, 99.3% (EtOH), was bought from Chemical Company (Iasi, Romania). An ammonium hydroxide (NH_4_OH) solution (28–30%) was purchased from Sigma-Aldrich (Merck KGaA, Darmstadt, Germany) EMSURE^®^ ACS, Reag. Ph Eur. All aqueous solutions were prepared with freshly distilled water.

### 2.2. Methods

#### 2.2.1. Synthesis and Functionalization of Silica Nanoparticles with PEI (SiO_2_-PEI NPs)

The preparation procedure for silica nanoparticles (SiO_2_ NPs) and silica nanoparticles functionalized with nitrile groups SiO_2_-CN NPs has been previously reported [18], and this was further modified to synthesize carboxyl functional group SiO_2_-COOH NPs on which a PEI brush was attached according to the procedures described below [18].

To prepare SiO_2_-COOH by acid hydrolysis of –CN functional groups, to a suspension of 10 g SiO_2_-CN NPs in approx. 50 mL water, an equal volume of H_2_SO_4_ 5M was added. The reaction mixture was refluxed under stirring at 700 rpm and 95 °C overnight. The particles were purified by centrifugation and washed with water until the supernatant had a neutral pH.

To prepare SiO_2_-PEI, we describe the coupling procedure between the SiO_2_-COOH and PEI: the aqueous suspension of SiO_2_-COOH NPs was centrifugated, separated by decantation, and the nanoparticles were resuspended in 10 mL of DMF. This procedure was repeated three times to ensure the complete removal of water, as it would react with the N,N′-dicyclohexylcarbodiimide (DCC) and hinder the coupling reaction. A suspension of 1.5 g of SiO_2_-COOH NPs in dry DMF was bubbled with Ar and stirred at room temperature for 15 min. An amount of 1.25 g DCC was added, and the mixture was left to stir for 10 min. Further, 2.7 g of branched polyethyleneimine was dissolved in 10 mL DMF and the solution was added dropwise to the reaction mixture. The mixture was left to react at 40 °C. The resulting particles were washed once with DMF, twice with ethanol and three times with water. The zeta potential ζ of the pristine silica nanoparticles and the functionalized ones was determined with a Zetasizer NanoZS (Malvern Panalytical Ltd., Malvern, UK) after multiple washing cycles with distilled water until the pH of the dispersed solution reached the pH value of the distilled water produced in our lab. The pH of the distilled water produced in our laboratory is pH = 5.7. 

#### 2.2.2. Microsphere Preparation via Pickering Emulsion Polymerization (P(t-BA)/SiO_2_-PEI NPs-Microsphere)

A Pickering emulsion was produced by first adding 30 mg of a benzoin methyl ether (BME) radical initiator to a 20 mL glass scintillator vial, followed by 1 mL of t-BA and 0.2 mL of DVB crosslinker. The mixture was left for 5 min for it to become homogeneous. Next, 5 mg of colloidal particles and 12 mL of water were added. The glass scintillator vial was then sonicated with a vortex for 1 min at 2000 rpm. 

#### 2.2.3. Preparation of 2D P(t-BA)/SiO_2_-PEI NPs and PVA/P(t-BA)/SiO_2_-PEI NP Films

To synthesize the 2D P(t-BA)/SiO_2_-PEI NP films, L-B experiments were carried with a Kibron Microtrough G1 equipped with a liquid/liquid o/w trough from Kibron Inc., Finland. Oil in water (o/w) emulsions, where the oil is represented by the t-BA containing the radical polymerization initiator (BME) monomer, were gently spread on the surface of the water contained in the Kibron LB trough with the help of a spoon spatula, where approximately 1 mL of Pickering emulsion was used. During spreading, the barriers of the L-B trough were fully open (100% area), and after film spreading, the barriers were closed, compressing the film until the area was approximately 10–20%. At this point, the barriers were closed, and the monomer film was exposed for 2 h to a UV lamp (wavelength = 365 nm, with 4 lamps, each with an intensity = 2.2 mW/cm^2^). After the polymerization, the polymer film was collected from the water surface either on a wire frame for further handling and processing or on an aluminum stub for scanning electron microscopy (SEM) investigations. 

To reinforce the obtained 2D P(t-BA)/SiO_2_-PEI NP film, a 2 mL mixture of a 3% PVA aqueous solution and glycerol (PVA/glycerol = 1:2 wt.) was poured over the membrane until it was completely and uniformly covered, and it was allowed to dry at room temperature so that a very thin film was formed.

#### 2.2.4. Measurement of Ion Extraction and Recovery Capacity of Polymer Absorbents 

The concentration of metal ions in the diluted supernatant and filtrate was determined using a UV-vis spectrophotometer model SP-UV1100 (DLAB Scientific Co., Ltd., Beijing, China). Calibration curves were first established based on the maximum absorption wavelength, λ_max_ = 812 nm for CuCl_2_ × 2H_2_O.

During the ion extraction process, which involves removing metal ions from a standard solution, a weighed amount of polymer adsorbent was mixed into 10, 15 or 20 mL of a stock solution with a predetermined concentration of 3 × 10^−2^ M for 12 h. The metal ion extraction capacity *q*_e_ (mg/g) was calculated with the following formula:

(1) $$q_{e}=\frac{\left(c_{i}-c_{e}\right)\;V}{m_{P}}$$

where *c*_i_ (mg/L) is the initial concentration of the stock solution or the contact solution, *c*_e_ (mg/L) is the concentration after extraction, *V* (L) is the volume of the sample, and *m*_P_ (g) is the dry mass of the adsorbent. 

For ion recovery, which refers to the recovery of metal ions from the polymer absorbent, the same polymer adsorbent used in extraction was immersed in 10, 15 or 20 mL of a 5% HCl solution. The samples were then left in this condition for approx. 12 h. The aqueous solution after recovery was analyzed using UV-vis, and the metal ion recovery capacity *q*_r_ (mg/g) was calculated by:

(2) $$q_{r}=\frac{c_{r}\;V}{m_{P}}$$

where *c*_r_ (mg/L) is the concentration of metal ions recovered from the particles, *V* (L) is the volume of the sample, and *m*_P_ (mg) is the dry mass of the adsorbent. 

### 2.3. Scanning Electron Microscopy (SEM)

The 2D films were investigated using a Verios G4 UC (Thermo Fischer Scientific Inc., Eindhoven, The Netherlands) scanning electron microscope (SEM) with a 5 keV beam energy using an Everhart–Thornley detector, with a beam spot of 50 pA. 

### 2.4. Optical Microscopy

The nanoparticles, the microspheres and films were analyzed with an IM-5FLD inverted fluorescence microscope (Optika Srl, Ponteranica, Italy) equipped with an 8 W XLED illumination source for sample analysis under transmitted light; 5 W LED excitation illumination sources at 470, 560, and 385 nm and UV, blue, and green filter sets for sample analysis in fluorescence mode; a color digital Camera Optica C-P6, 6.3 MP; and Optica Pro View (Optika Srl, Ponteranica, Italy) software for image capturing and processing (https://www.optikamicroscopes.com/optikamicroscopes/product/optika-software/, accessed on 1 April 2024). All samples were analyzed with 4× and 10× magnification objectives in transmitted illumination mode. For capturing iridescence, the samples were illuminated with a light source placed sideways from the objective axis.

## 3. Results and Discussions

In this work, we employ tert-butyl acrylate (t-BA) as the oil phase to create an oil-in-water (o/w) Pickering emulsion stabilized by SiO_2_-PEI NPs. By further compressing the t-BA/water interface in the L-B trough, we achieve an enhanced control over particle interfacial assembly by using the L-B and Pickering emulsion polymerization methods that materialize into an asymmetrically nanostructured 2D thin poly(t-butyl acrylate) (P(t-BA)) film. We further demonstrate that when silica nanoparticles are functionalized with interfacial ligands, such as polyethyleneimine brushes, these 2D thin nanostructured films could be deployed in various applications, for example, metal-ion extraction and recovery from water. These films can be further re-enforced with a polyvinyl alcohol/glycerol film that provides excellent sturdiness and flexibility. In addition to the creation of metal ion adsorbent the combined L-B and PEmPTech can also be applied to producing 2D nanophotonic monolayer crystals [2,3,24], mitigating the fragility of the resulting monolayers due to reduced inter-particle cohesion that disrupts the crystal-like ordering of the monolayers. In this work, we suggest this possibility by producing 2D monolayer crystals by spreading a Pickering emulsion of a monomer on the surface of water in an L-B trough. Due to a complex mechanism of surface re-arrangement of the nanoparticles at the oil–water interfaces, a continuous monolayer of silica nanoparticles is produced, which upon polymerization evolves into a 2D nanostructured P(t-BA) film with a highly organized self-assembled monolayer of silica nanoparticles that exhibits iridescence and a hint of structural colors, which are, as already mentioned above, most commonly found in nature and biomimetic materials [22,23].

### 3.1. Synthesis SiO_2_-PEI NPs

**Figure 1:**
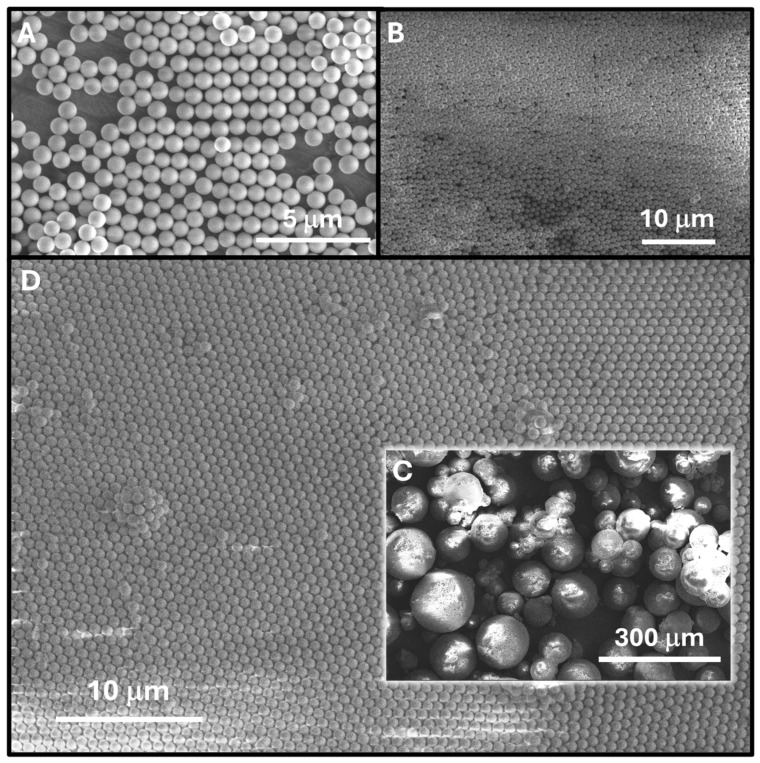
SEM images of the obtained SiO_2_-PEI NPs showing (**A**) the tendency of these nanoparticles to self-assemble and (**B**) the tendency of these nanoparticles to self-assemble at larger scale in a cast film upon removal of water. SEM images of the polymer microspheres obtained (**C**) exhibit a nanostructured surface with SiO_2_-PEI NPs at their surface perfectly self-assembled into a compact close-packed arrangement (**D**).

**Figure 3:**
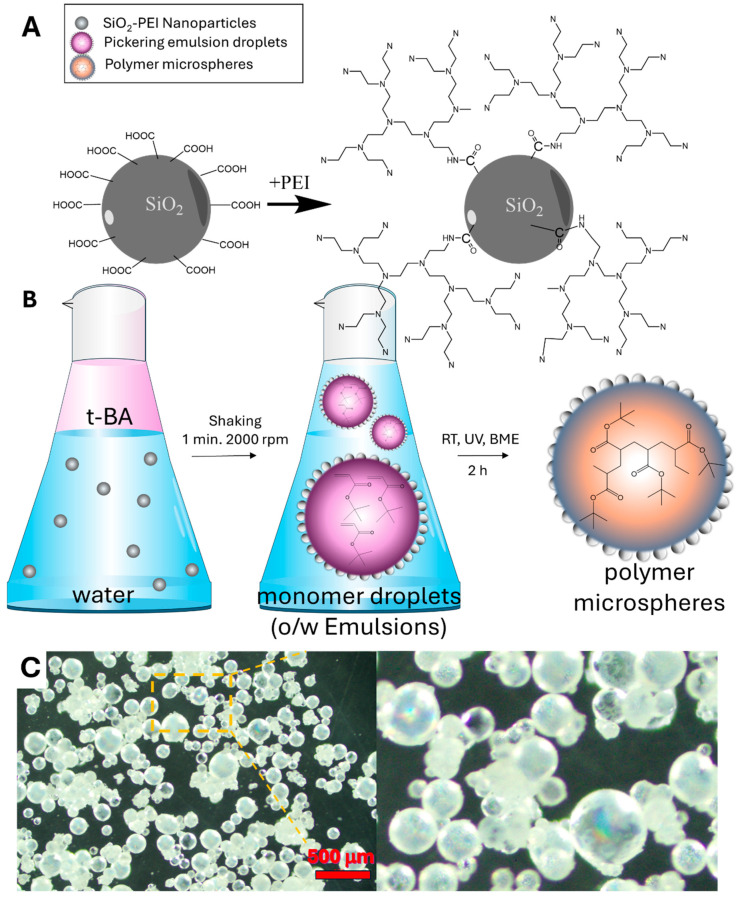
Reaction scheme for the surface coupling of b-PEI on the surface of the SiO_2_-COOH NPs (**A**). (**B**) Schematics showing the process of Pickering emulsion preparation from two immiscible phases, the liquid organic phase that is the monomer and the water phase containing a suspension of silica nanoparticles, followed by its conversion into an o/w emulsion and polymerization. (**C**) Optical microscope images of the P(t-BA) microspheres, showing iridescence due to diffraction of the light from the ordered self-assembled monolayers of silica nanoparticles at its surface.

The silica nanoparticles were synthesized according to procedures we have previously reported [18]. The average diameter of the obtained SiO_2_-NPs was 737 ± 4 nm. The SEM image of these NPs is presented in Figure 1A,B. Further, the SiO_2_-NPs were functionalized with TESPN to generate nanoparticles with nitril groups on their surface (SiO_2_-CN NPs). Further, the nitril groups were transformed by hydrolysis in acidic conditions in the carboxyl groups, so we have generated carboxyl-bearing silica nanoparticles (SiO_2_-COOH NPs). Next, we coupled branched polyethyleneimine b-PEI on the surface of these SiO_2_-COOH NPs with the help of the DCC coupling agent to obtain SiO_2_-PEI NPs, according to the reaction scheme presented in Figure 3A. The evolution of the zeta potential with the surface chemical reaction is given in Table 1, and while the starting NPs had a negative surface potential at normal pH, the zeta potential of the SiO_2_-CN NPs slightly decreased due to the excess negative charge brought by the nitrile groups. The zeta potential increases with the hydrolysis of the nitrile functional groups and their conversion to carboxyl functional groups, SiO_2_-COOH NPs, which are partially protonated at neutral pH = 5.7. SiO_2_-PEI NPs have a positive surface potential, which is supporting evidence that the surface functionalization has been successful. 

**Figure 2:**
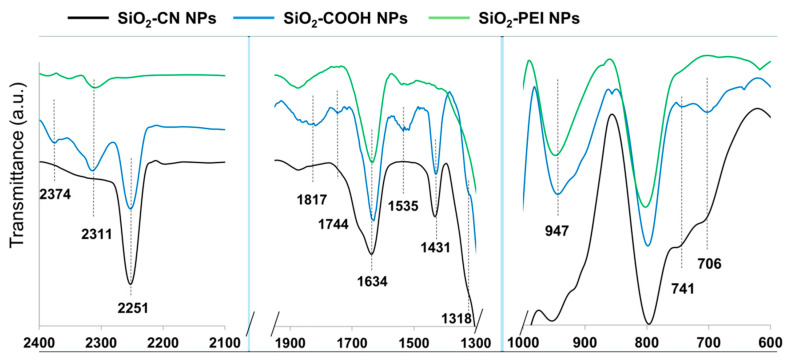
FTIR spectra of the SiO_2_-CN NPs, SiO_2_-COOH NPs and of the SiO_2_-PEI NPs.

The FTIR spectra of the silica nanoparticles are given in Figure 2, and Figure S1 shows the chemical modification following the surface reactions with functionalization agents as follows. Structural vibration modes of the silica nanoparticles are typically observed at 1100 cm^−1^ and are associated with asymmetric Si-O-Si vibrations and symmetric Si-O-Si vibrations at 790 cm^−1^ as well as Si-O-Si deformation vibrations at 471 cm^−1^, which are also typically observed in cyclic siloxanes. Further, also characteristic to silica nanoparticles are the Si-OH stretching vibrations observed at 947 cm^−1^ and deformation vibrations at 1634 cm^−1^. The broad bands observed around the value of ≈3400 cm^−1^ are associated with water bound by hydrogen bridges and the stretching vibrations of the Si-OH bond isolated or linked by hydrogen bridges [18,25].

For the SiO_2_-COOH NPs, the FTIR spectrum shows the disappearance of the -C≡N band at 2251 cm^−1^, which was found in the starting SiO_2_-CN NPs and even remnant in the SiO_2_-COOH NPs after hydrolysis, signaling that PEI modification led to the disappearance of this band. In addition, the successful chemical modification of the surface of the NPs is confirmed by the disappearance of the characteristic vibrations of the carboxyl groups at 1535 cm^−1^, 1744 cm^−1^ and 1817 cm^−1^. In addition, the peaks at 2872 cm^−1^ and 2901 cm^−1^ overlap after modification of the nanoparticles with PEI, and we believe this is due to the strong hydration of the PEI corona and appearance of a very broad and intense hydrogen bond band.

### 3.2. Synthesis of the P(t-BA)/SiO_2_-PEI NPs-Microspheres

To synthesize P(t-BA)/SiO_2_-PEI NPs-Microspheres, we have first generated a stable Pickering emulsion from the monomer t-BA and water containing SiO_2_-PEI NPs that stabilizes the emulsions, according to the scheme presented in Figure 3B. The Pickering emulsions are stabilized due to the interfacial adsorption of the silica nanoparticles at the oil–water interface with the formation of a self-assembled monolayer. The monolayer of nanoparticles acts like a shield preventing the coalescence of the oil droplets [18,26]. Thus, these special structures, the liquid Pickering emulsion droplets covered by a shield of self-assembled monolayer of nanoparticles, are often referred to as “colloidosomes” and will be referred to in the same way in the current work.

The emulsion generated was stable and could be thus polymerized at room temperature (RT) under exposure to UV radiation, and this way, all the monomer droplets/colloidosomes formed in the emulsion are converted into P(t-BA) microspheres, see Figure 1C. The surface of these microspheres is nanostructured due to the trapping of the self-assembled monolayer of nanoparticles. The SEM images in Figure 1D reveal a highly organized structure of these nanoparticles that pack in a hexagonal closed-packed (HCP) arrangement on the large surface area of the microspheres, forming a perfectly self-assembled film as compared to a less organized structure in the cast film Figure 1A,B. The optical microscope images of the polymer microspheres, Figure 3C, reveal multicolored iridescent beads due to the diffraction of light from the ordered structure of the self-assembled monolayer at the surface of the microspheres, a well-known phenomenon observed in photonic crystals or highly ordered crystalline monolayers of nanoparticles [27].

### 3.3. Synthesis of the Langmuir–Blodgett 2D P(t-BA)/SiO_2_-PEI NP Film

**Figure 4:**
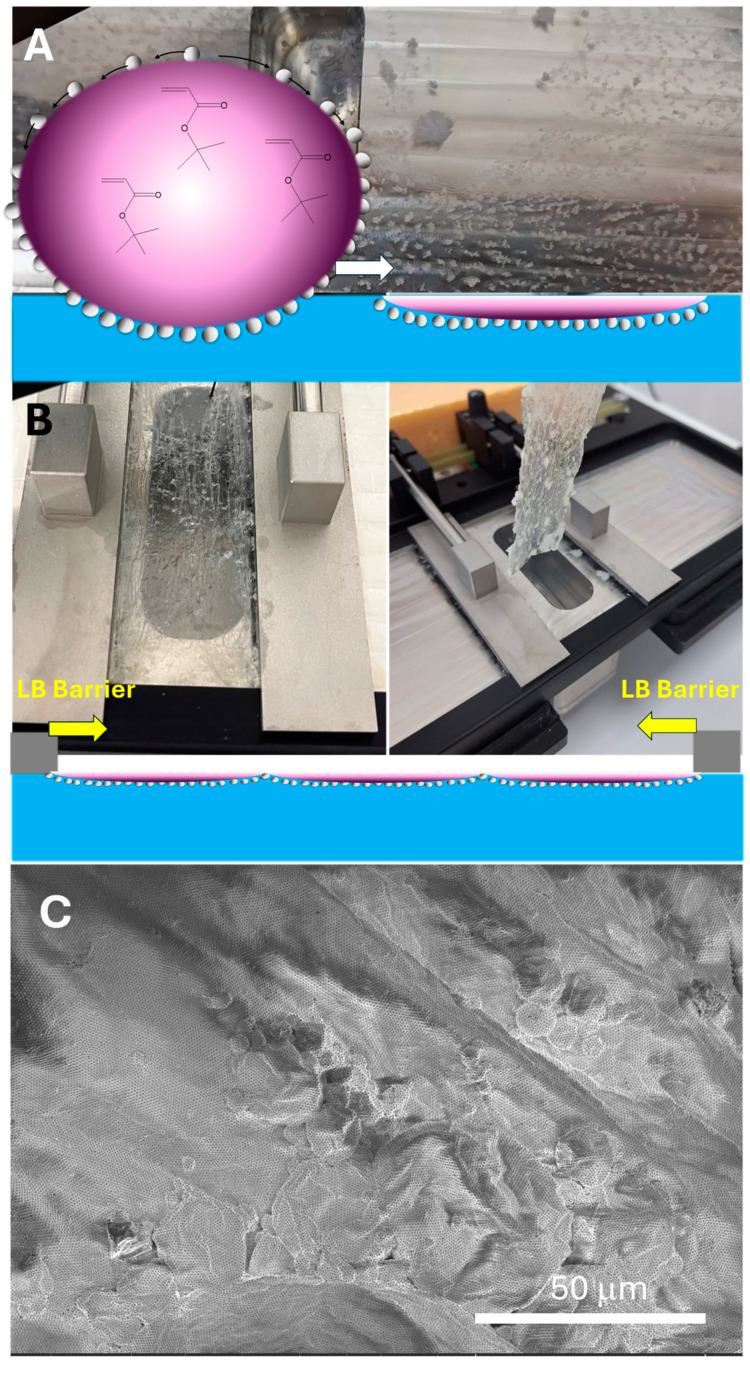
(**A**) The spreading of the Pickering emulsion on the water surface of the Langmuir–Blodgett trough. The cartoon depicts the spreading of the droplet on the water surface with the re-arrangement of the SiO_2_-PEI NPs at the monomer/water interface. The background photograph shows the initial shape of the colloidsomes on the surface of water. (**B**) The closing of the L-B barriers and the compression of the film followed by polymerization under UV light. The background images show the 2D P(t-BA)/SiO_2_-PEI NP film obtained after polymerization. (**C**) SEM images of the 2D P(t-BA)/SiO_2_-PEI NP film exhibiting a wavy structure and circular domains of partially integrated colloidosomes.

The process of forming Janus 2D P(t-BA)/SiO_2_-PEI NP films from the Pickering emulsions in an L-B trough is similar to the one we have previously reported [26]. Briefly, the Pickering emulsion droplets (colloidosomes) were added onto the surface of a Langmuir–Blodgett trough with a spatula. The emulsion droplets exhibit an initially highly energic motion on the surface of the water until they break, forming a film, Figure 4A. We presume that the energic motion is due to re-arrangement of the interface due to a competitive wetting process, such that the SiO_2_-PEI NPs, being hydrophilic, are wetted by water; at the same time these are also wetted by the monomer, the SiO_2_-PEI NPs also remain attached to the monomer droplet surface. As we will see next, the SiO_2_-PEI NPs exclusively occupy the monomer/water interface. A similar phenomenon was also observed in the case of silica nanoparticles bearing different functional groups, such as glycidyl, and was observed for other particles bearing surface functional groups with a higher component of the dispersive surface energy [26]. The monomer droplet/water interfacial tension is stabilized by the presence of the nanoparticles. Thus, we hypothesize that the energic motion of the monomer droplet is due to Marangoni gradients acting upon the monomer colloidosome, as well as the capillary forces due to initial wetting of the self-assembled SiO_2_-PEI NP monolayer at the surface of the colloidosomes by water. 

Next, after approximately 1 mL of Pickering emulsion was spread on the water surface, the L-B barriers were compressed such that the available surface area decreased from initially 100% to ca. 20%; these values refer to the absolute area of the trough and were displayed on the graph of the isotherm during compression. When the barriers of the trough were fully open, the available water surface area was 100%, and when the barriers were closed, this decreased to approximately 20%. Through trial and error, we adjusted the volume spread to 1 mL to obtain a reasonable match between the area occupied by the nanoparticles and the area of the trough at full compression, as verified via SEM, Figure 4. After the barriers close, the polymerization begins by exposing the film to a UV lamp for 2 h. After the polymerization, see Figure 4B, the polymer film is collected from the water surface with a tweezer for further handling and processing or on an aluminum stub for scanning electron microscopy (SEM) investigations. The cartoon in Figure 4B already suggests that the film thickness fluctuation originates from the boundaries of broken colloidosomes spread on the surface of the water. When the surface is supersaturated with the Pickering emulsions, the colloidosomes will not break, and will only be partially integrated into the film, as shown in the SEM images of Figure 4C. 

**Figure 5:**
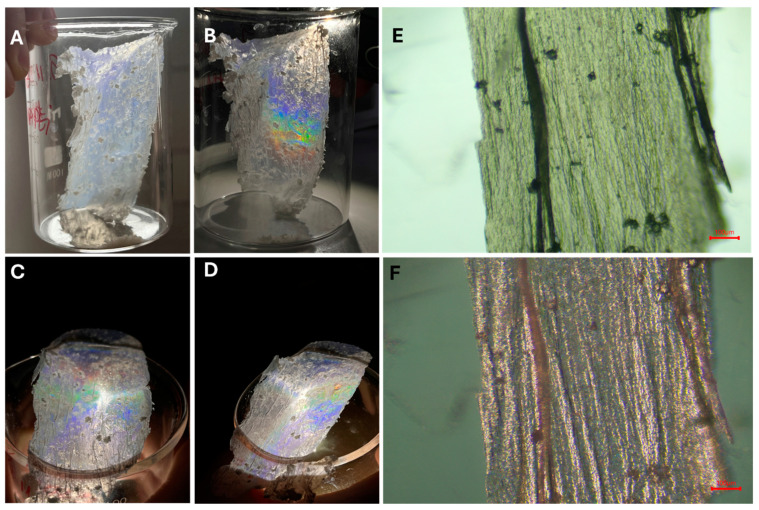
(**A**) Photograph of the highly organized crystalline 2D P(t-BA)/SiO_2_-PEI NP monolayer after drying; (**B**–**D**) photographs of the film showing multiple colors while being illuminated with light from a smartphone; (**E**) optical microscope image of the 2D film; and (**F**) the red structural color of the film as appears upon illumination at an ≈60° off-normal objective axis.

After drying, the 2D P(t-BA)/SiO_2_-PEI NP film obtained appears to be sufficiently rigid for careful handling with tweezers, as shown in Figure 5A. Interestingly, the membrane exhibits iridescence, showing rainbow colors due to the Bragg diffraction of light upon illumination of the membrane with the lamp from a smartphone at certain incident angles, see Figure 5B–D, which resembles the properties of photonic crystals or a monolayer crystal of nanoparticles, also prepared via the Langmuir–Blodgett technique [2,24,27]. The optical microscope images in Figure 5E,F further show the more intricate details of the 2D P(t-BA)/SiO_2_-PEI NP film, with a hint of red structural color. In contrast, while these were prepared by the transfer of a fragile monolayer of nanoparticles onto a solid substrate by dip-coating, which may disrupt their organization, the remarkable advantage in the current case is that the great degree of ordering achieved by L-B technique is preserved due to the polymerization of the monomer. Thus, it is this organization that leads to the iridescent structural colors of the films, with potential applications in various fields. We have thus demonstrated a facile method for the fabrication of such 2D highly organized crystalline monolayers supported on a thin P(t-BA) film, which, to the best of our knowledge, is being presented here for the first time. 

**Figure 6:**
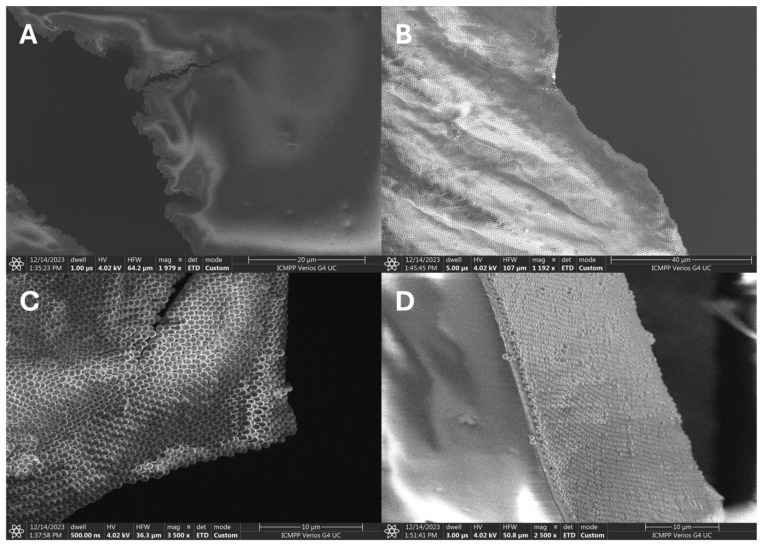
SEM images of the 2D highly organized crystalline monolayer of SiO_2_-PEI NPs, taken from (**A**) the back side of the film, the polymer–air interface; (**B**) the front side of the film, the polymer–water interface, where nanoparticles can be seen, in a long-range, high degree of ordering; (**C**) further zooming on the membrane shows the almost perfectly crystalline degree of ordering for the 2D film and (**D**) the film in the cross-section.

The SEM images presented in Figure 6 reveal an asymmetric Janus-like characteristic of the 2D P(t-BA)/SiO_2_-PEI NP film. On one side of the film, Figure 6A, there are no particles to be observed, and this is the side of the film (the back side) that is not in contact with water but with air, the polymer/air interface. On the other side of the film, Figure 6B,C, a highly organized monolayer of self-assembled SiO_2_-PEI NPs can be observed, and this comes from the polymer/water interface of the film (the front side). Figure 6B shows a large area of ordering of the monolayer of nanoparticles, with an almost perfect hexagonal-closed crystalline packing. 

The SEM images in Figure 6D shows the cross-section of the 2D film, where the asymmetric Janus-like characteristic can be clearly observed, with the SiO_2_-PEI NPs populating only one side of the film (on the polymer/water interface), while the other side is completely void of nanoparticles. In addition, the thickness of the P(t-BA) film ranges between 500 and 1200 nm. Figure 6D provides a snapshot of the high degree of ordering of the NPs on large areas of the film surface; for example, rows of ordered nanoparticles can be seen running from the edge of the cross-section deep into the visual field of the film for tens and hundreds of microns. Such ordered structures have been produced in our group and reported previously in Pickering emulsions [18,28]. 

One disadvantage of the film is its relative fragility; the thin 2D monolayer breaks very easily if not carefully manipulated. Thus, to provide better support and strengthen the film, we have reinforced it by pouring 2 mL of an aqueous solution of a PVA/glycerine mixture onto approximately an 8 cm^2^ area of the side of the film free from nanoparticles. This produces a flexible PVA/P(t-BA)/SiO_2_-PEI NP film that is sturdier and can be easily handled for further use in applications; for example, it can be deployed in multiple cycles of adsorption and desorption of metal ions from aqueous solutions. The photograph of the obtained film is presented in Figure S2. 

### 3.4. Application of Thin 2D P(t-BA)/SiO_2_-PEI NP Films for Ion Adsorption 

The 2D P(t-BA)/SiO_2_-PEI NP film was next deployed to test its capacity for adsorption and desorption of metal ions from water samples. Its adsorption capacity was compared with that of the P(t-BA)/SiO_2_-PEI NPs-Microsphere, SiO_2_-PEI NPs and PVA/P(t-BA)/SiO_2_-PEI NP films. The active components of binding and capturing metal ions from water are the SiO_2_-PEI NPs through the PEI layer at their surface. According to our previous studies [29,30], the PEI layer is rather non-specific and capable of binding a variety of different metal ions, albeit with different affinities. However, the use of nanoparticles in real applications for ion extraction is not desired as they may escape into the surrounding medium, posing a potential environmental threat themselves. However, silica nanoparticles are generally considered neutral to the environment, as they are already largely encountered in the current form in nature [31]. Thus, in this study, we measure the evolution of the adsorption capacity of the SiO_2_-PEI NPs in different states in powder form as self-assembled monolayers on the surface of microspheres in a 2D L-B film and in an imbedded PVA film. The capacity of these materials was tested for adsorption of Cu(II) metal ions from aqueous solutions. As already alluded to, the extraction capacity *q_e_* refers to the extraction of metal ions from a solution containing the metal ion, and in this case, the difference between the concentration of the initial solution and that of the solution after being in contact with the adsorbent for 12 h was measured according to Equation (1). On the other hand, *q_r_* refers to the recovery of metal ions from the material using an acidic solution, and the recovery capacity of metal ions was calculated according to Equation (2).

**Figure 7:**
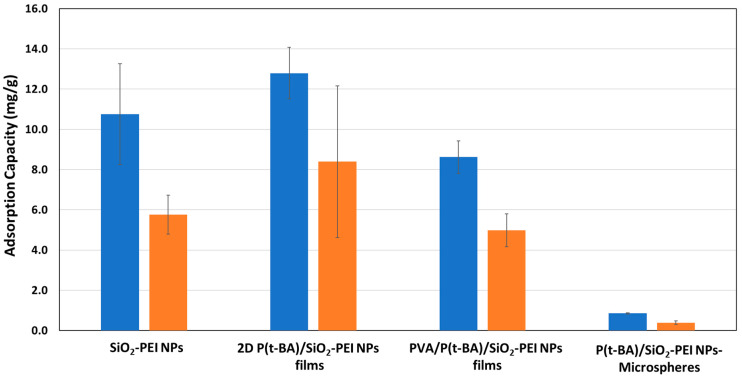
The extraction (blue bars) and recovery capacity (orange bars) of Cu(II) ions for SiO_2_-PEI NPs, 2D P(t-BA)/SiO_2_-PEI NP films, P(t-BA)/SiO_2_-PEI NPs-Microspheres, PVA/P(t-BA)/SiO_2_-PEI NP films.

The calculated adsorption capacities, *q_e_* and *q_r_*, for Cu(II) are provided for each sample in Figure 7. Several conclusions can be drawn from the presented data. First, the adsorption capacities decrease in the following order: 2D P(t-BA)/SiO_2_-PEI NP film (*q_e_* = 8.4 mg/g, *q_r_* = 12.8 mg/g) > SiO_2_-PEI NPs (*q_e_* = 5.8 mg/g, *q_r_* = 10.8 mg/g) > PVA/P(t-BA)/SiO_2_-PEI NP films (*q_e_* = 5.0 mg/g, *q_r_* = 8.6 mg/g) > P(t-BA)/SiO_2_-PEI NPs-Microspheres (*q_e_* = 0.4 mg/g, *q_r_* = 0.9 mg/g). Given that the SiO_2_-PEI NPs are the only active component for binding the metal ions, one might expect them to exhibit the highest adsorption capacity. However, the results suggest otherwise. This discrepancy could be partially explained by the close numerical results between 2D P(t-BA)/SiO_2_-PEI NP films and SiO_2_-PEI NPs, which fall within the experimental error margin of the measurement. The large experimental error, especially in the case of the 2D P(t-BA)/SiO_2_-PEI NP film, is due to the difficulty in handling an ultrathin sample film, which was solved by reinforcing it with PVA. The further decrease in the capacity of adsorption, significant for the P(t-BA)/SiO_2_-PEI NPs-Microsphere and only slightly for the PVA/P(t-BA)/SiO_2_-PEI NPs, can be explained by addition of inert materials, P(t-BA) and PVA, which theoretically absorb little or no Cu(II) ions. Nonetheless, it is notable that the 2D P(t-BA)/SiO_2_-PEI NP film and the PVA/P(t-BA)/SiO_2_-PEI NP film exhibit a better adsorption capacity than that of nanoparticles and could potentially be used as an adsorbent on water surfaces for environmental remediation [31], which also poses the risk of losing expensive adsorbent nanomaterials. Therefore, the 2D P(t-BA)/SiO_2_-PEI NP films or PVA/P(t-BA)/SiO_2_-PEI NP films provide similar adsorption capacities to that of the nanoparticle powder but have an improved sample handling and control. To further emphasize the ease of handling, especially for the PVA/P(t-BA)/SiO_2_-PEI NP films, we provide an image of the film in Figure S2.

The current results prove that the adsorption capacity for Cu(II) metal ions makes these 2D thin nanostructured films extremely competitive to other nano-engineered material adsorbents [32,33]. For a literature comparison, Janus nanoparticles modified with PEI brushes have shown an adsorption capacity for Cu(II) ions of 6 mg/g [29], which aligns well with the capacity of SiO_2_-PEI NPs (*q_e_* = 5.8 mg/g, *q_r_* = 10.8 mg/g) in this study. For the wax colloidosomes generated with the same Janus nanoparticles, as reported by Pauli [29], the adsorption capacity for Cu(II) was ≈0.5 mg/g, which is comparable with that of the P(t-BA)/SiO_2_-PEI NPs-Microsphere (*q_e_* = 0.4 mg/g, *q_r_* = 0.9 mg/g) obtained in this work. Conversely, Tan et al. [30] have reported an adsorption capacity for Cu(II) for polystyrene nanoparticles modified with PEI of between 20 and 40 mg, which is at least double that of the SiO_2_-PEI NPs reported here. To our knowledge, there are no similar reports for 2D membranes of adsorbents thus far, highlighting the novelty of the current combined L-B and Pickering emulsion technologies to produce a 2D membrane of nanoparticles capable of metal ion adsorption.

In addition, the fact that the nanoparticle layer is found in a highly organized form on the surface of the P(t-BA) film or microspheres makes it attractive for a combination of applications, such as nanophotonic structural colors [22], photonic pigments [1], biomimetics [23], or sensors. This current method of manufacturing 2D, thin, supported monolayer crystals combining the L-B method and Pickering emulsions can be used without adaptation for other types of nanoparticles, rods, and nanostructures.

## 4. Conclusions

This investigation successfully synthesized a 2D thin film featuring a highly ordered monolayer of PEI-functionalized silica nanoparticles, achieved via a novel integration of Langmuir–Blodgett and Pickering emulsion techniques. This film demonstrates significant capabilities in metal ion adsorption, particularly Cu(II) ions, and exhibits iridescence due to its structural order. The adsorption capacity analysis highlights the film’s competitive performance compared to existing materials. The additional reinforcement with a PVA/glycerol layer addresses the film’s fragility, enhancing its practical applicability for environmental applications. This modification does not compromise the film’s functional properties but rather expands its utility, allowing for more robust handling and operational flexibility in real-world applications.

Future research should aim to refine the film’s adsorption efficiency; continue to explore into the mechanistic aspects of its ion exchange, adsorption, and recovery properties; and explore its adaptability to other pollutants and metal ions. The photonic characteristics of the film also present a new direction for research, suggesting potential cross-disciplinary applications, approached with multifunctional materials.

In sum, this work advances the field of nanoengineered materials with its methodological innovation and provides a versatile platform for generating materials capable of addressing pressing environmental challenges, such as metal ion removal and recovery for wastewater treatment and environmental remediation.

## Figures and Tables

**Table 1:** Zeta potential values of the SiO_2_-NPs at pH = 5.7 before and after each functionalization step.

Functional Group at the Surface	ζ-Potential [mV]
SiO_2_ NPs	−48.7 ± 1.4
SiO_2_-CN NPs	−51.3 ± 0.5
SiO_2_-COOH NPs	−37.5 ± 0.8
SiO_2_-PEI NPs	+48.9 ± 0.7

## Data Availability

The data presented in the study are available from the corresponding author.

## Supplementary Materials

The following are available online at https://www.mdpi.com/article/10.3390/nano14090809/s1, Figure S1: FTIR spectra of the nanoparticles; Figure S2: photograph of the PVA/P(t-BA)/SiO_2_-PEI NP films.
