# Supplementary material for: Interfacing Langmuir–Blodgett and Pickering Emulsions for the Synthesis of 2D Nanostructured Films: Applications in Copper Ion Adsorption
Source: Nanomaterials (Basel). 2024 May 6;14(9):809. doi: 10.3390/nano14090809 (PMC11085534; doi:10.3390/nano14090809)
Supplement: Supplementary file 1 [file nanomaterials-14-00809-s001.zip › nanomaterials-2975171-supplementary.pdf]

Supporting Information

Interfacing Langmuir-Blodgett and Pickering Emulsions for the Synthesis of 2D Nanostructured Films: Applications in Copper Ion Adsorption

Andrei Honciuc*, Oana-Iuliana Negru, Mirela Honciuc

"Petru Poni" Institute of Macromolecular Chemistry, 41A Gr. Ghica Voda Alley, Iasi, 700487, Romania

* Correspondence: honciuc.andrei@icmpp.ro

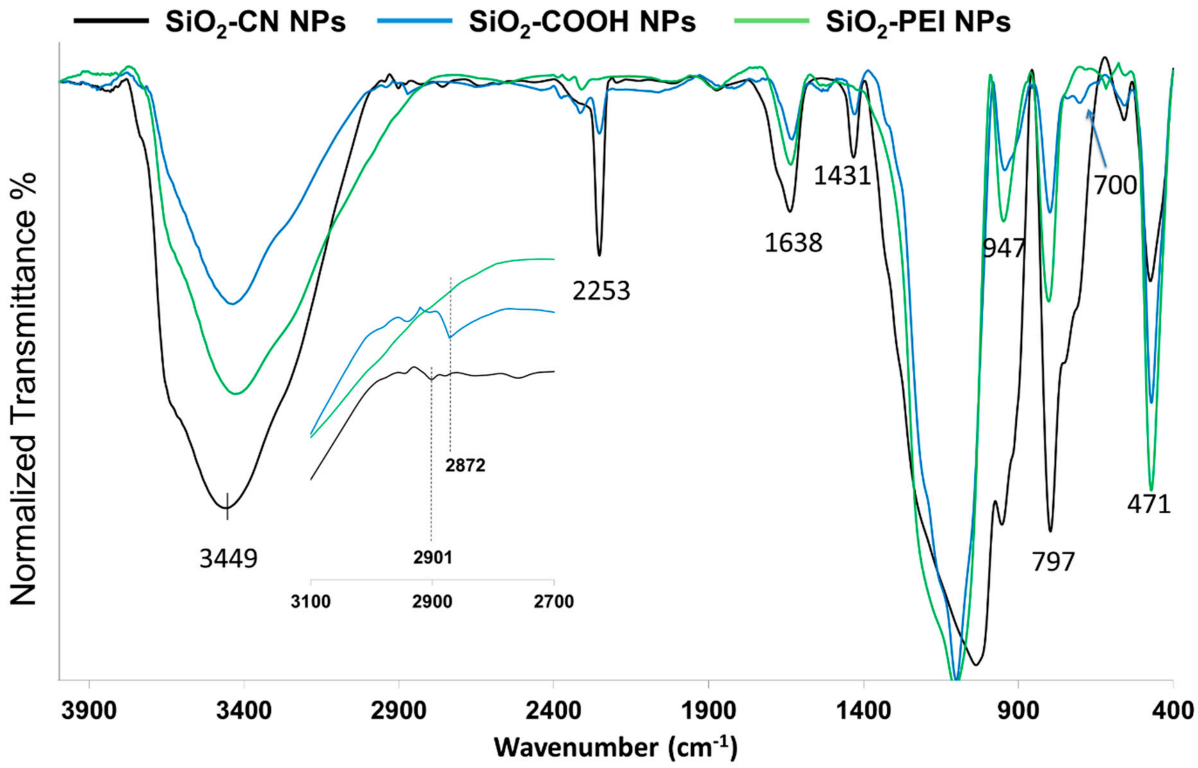

Figure S1. Normalized intensity FTIR spectra of the SiO_2-CN NPs, SiO_2-COOH NPs and of the SiO_2-PEI NPs.

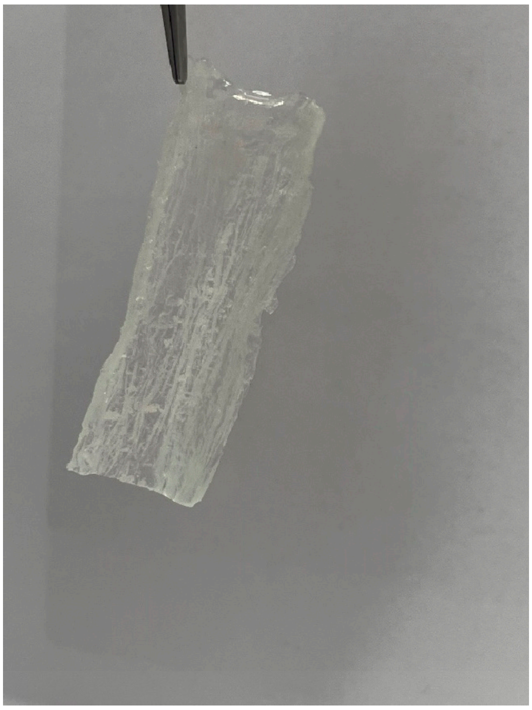

Figure S2. Image of the PVA/P(t-BA)/SiO_2-PEI NPs films taken after performing the Cu(II) adsorption and desorption experiments, showing that it is sturdy and flexible and can be easily manipulated.
